# Magnetic resonance imaging of the female pelvis after Cesarean section: a pictorial review

**DOI:** 10.1186/s13244-020-00876-5

**Published:** 2020-05-27

**Authors:** Monika Bekiesinska-Figatowska

**Affiliations:** Department of Diagnostic Imaging, Institute of Mother and Child, Kasprzaka 17a, 01-211 Warsaw, Poland

**Keywords:** Cesarean section (C-section), Pelvis, Magnetic resonance imaging (MRI)

## Abstract

The rate of Cesarean sections (C-sections) in Poland increased from 21.7% in 2001 to 43.85% in 2017 even though the Polish Society of Gynecologists and Obstetricians highlights the negative consequences of C-section for both mother and child and recommends to make every possible effort to reduce its percentage, following the World Health Organization recommendations. There is a long list of possible complications related to the uterine scar after C-section, including uterine scar dehiscence, uterine rupture, abdominal and pelvic adhesions, uterine synechiae, ectopic pregnancy, anomalous location of the placenta, placental invasion, and—rarely—vesicouterine or uterocutaneous fistulas. Ultrasound (US) remains the first-line modality; however, its strong operator- and equipment dependence and other limitations require further investigations in some cases. Magnetic resonance imaging (MRI) is the second-line tool which is supposed to confirm, correct, or complete the sonographic diagnosis thanks to its higher tissue resolution and bigger field of view. This article will discuss the spectrum of C-section complications in the MR image-rich form and will provide a systematic discussion of the possible pathology that can occur, showing comprehensive anatomical insight into the pelvis after C-section thanks to MRI that facilitates clinical decisions.

## Key points

- Cesarean section (C-section) is a popular delivery technique which—being necessary for certain conditions—can lead to a considerable percentage of complications.
- Ultrasound (US) is the first-line imaging tool in the detection of these complications.
- Acute intraoperative and postoperative complications are addressed with the use of computed tomography (CT) if diagnostic imaging is necessary.
- Magnetic resonance imaging (MRI) reveals consequences and complications of C-section in a targeted pelvic examination but also incidentally in the L-S spine examination.

## Background

The rate of Cesarean sections (C-sections) in Poland increased from 21.7% in 2001 to 43.85% in 2017 even though the Polish Society of Gynecologists and Obstetricians highlights the negative consequences of C-section for both mother and child; recommends to make every possible effort to reduce its percentage, among healthy primiparous women with uncomplicated pregnancies in particular; and determines the optimum indications for C-sections [1]. The World Health Organization (WHO) recommends a C-section rate of 10–15%, only when medically necessary [2]. There are countries with higher percentage of C-sections than Poland, e.g., Turkey (53%), Korea (45.2%), Mexico (45%), and Chile (45%), but we have overtaken such countries as Italy (36%) or the USA (32%) [3–5]. The problem is widespread all over the world. The latest available data from the Organisation for Economic Co-operation and Development (OECD) indicate that out of the 29 countries surveyed (25 European countries, Israel, Canada, New Zealand, and Korea), it is only Israel that has a percentage of Cesarean sections within the WHO recommended limits (14.8%) [5].

The indications for C-section differ among the countries; they can be found and are discussed with varying degrees of detail, for example, in the National Institute for Health and Care Excellence (NICE) Guidance: Cesarean section, last updated in September 2019 [6]. The short and concise recommendations can be found in German-speaking countries as follows:

1. Absolute indications
  - Absolute disproportion: small maternal pelvis, making vaginal birth impossible
  - Chorioamnionitis (amniotic infection syndrome): infection of the placenta and possibly of the fetus, requiring immediate delivery
  - Maternal pelvic deformity making vaginal birth impossible
  - Eclampsia and HELLP syndrome
  - Fetal asphyxia or fetal acidosis
  - Umbilical cord prolapse between the head of the fetus and the vaginal opening
  - Placenta previa
  - Abnormal lie and presentation
  - Uterine rupture
2. Relative indications
  - Pathological cardiotocography (CTG)
  - Failure to progress in labor (prolonged labor, secondary arrest)
  - Previous cesarean section

Cesarean delivery on maternal request without any medical indication is considered a separate indication [7].

In the majority of cases, skin incision is Pfannenstiel incision (transverse suprapubic cut). The uterine incision may be classical (midline vertical) or—most commonly performed—transverse just above the bladder edge (lower (uterine) segment Cesarean section (LSCS)).

It is hypothesized that the surgical technique of uterine incision closure is the most important determinant of C-section defect formation. The appropriate suture of the myometrial edges (in two layers of non-locking continuous sutures without undue tightness and minimal inclusion of decidua) allows their best apposition without devascularization, as ischemic necrosis of the myometrial tissue is considered as responsible for the formation of C-section defect, scarring, and adhesions [8].

There is a long list of possible maternal and fetal complications related to the uterine scar after C-section, including uterine scar dehiscence, uterine rupture, abdominal and pelvic adhesions, uterine synechiae, ectopic pregnancy, anomalous location of the placenta, placental invasion, and—rarely—vesicouterine or uterocutaneous fistulas [9, 10]. Other maternal complications include (chronic) pelvic pain, (chronic) incision site pain, dysmenorrhea, abnormal vaginal bleeding, endometriosis, and reduced future fertility [11]. The risk of serious maternal morbidity (placenta previa, accreta/increta/percreta, uterine dehiscence or rupture, postpartum hemorrhage, blood transfusion, bladder injury), duration of the operation and of hospital stay, and the number of admissions to intensive care unit increase with increasing numbers of previous Cesarean sections [12].

**Fig. 1:**
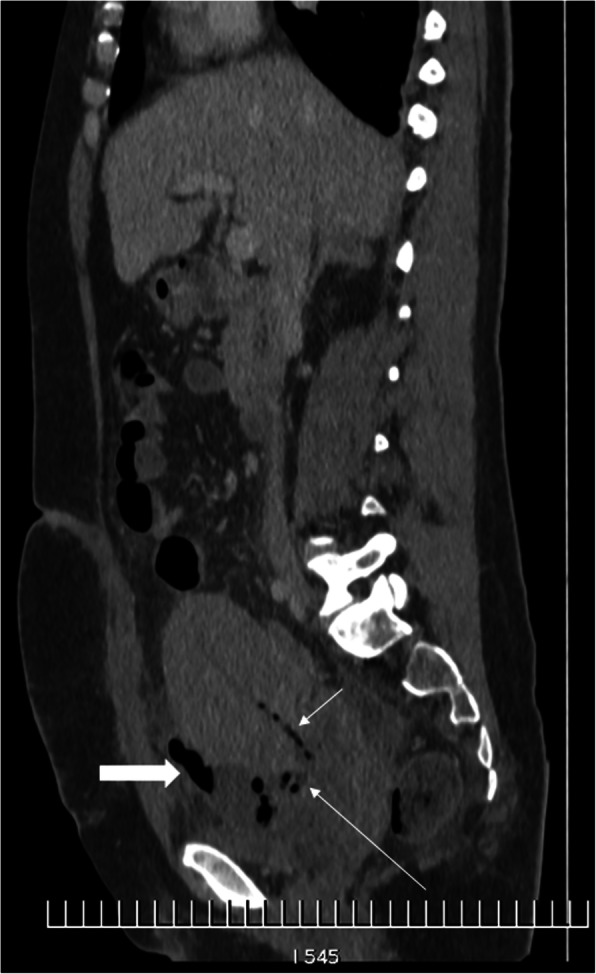
A 33-year-old woman 6 days after repeat C-section in 2nd pregnancy. CT is performed due to fever, pain, left costovertebral angle tenderness, and increasing serum level of C-reactive protein (485 mg/l) and procalcitonin (6.7 ng/ml), with a suspicion of renal colic or endometritis. CT revealed gas within the C-section incision in the uterine wall (long arrow) and in the uterine cavity (short arrow), as well as an abscess in front of/under the uterus (thick arrow), shifting the bladder to the left. Two hours later, the patient was operated on, and the abscess was evacuated

The complications require—among others—diagnostic imaging. Ultrasound (US) remains the first-line modality; however, its strong operator and equipment dependence and other limitations require further investigations in some cases. Pelvic MRI is the second-line tool which is supposed to confirm, correct, or complete the sonographic diagnosis [13]. Computed tomography (CT), as in most gynecological-obstetric situations, is of limited value, and the ratio of potential benefits to the burden of ionizing radiation and iodine contrast agent does not justify the use of CT except for acute complications like active arterial bleeding in case of postpartum hemorrhage [14] or other acute maternal complications (Fig. 1) [11]. Therefore, it is important to remember the advantages of MRI over CT. An interesting review of post-C-section complications in various imaging methods has been published lately and discusses them in detail [15]. In this review—with a different focus—the author presents her own experience in MR imaging of more C-section complications, mainly the late ones, thus providing the readers with new useful information. The proposed scanning protocol of pelvic MRI is shown in Table 1.

**Table 1:** The proposed scanning protocol of pelvic MRI

Sequence	Projection	TR (ms)	TE (ms)	FOV (cm)	Slice thickness/interslice gap (mm)	Matrix	NEX
FRFSE/T2	sag, ax	5000	100	24 × 24	5.0/1.5	512 × 256	4
FRFSE/T2+fs	cor	5640	102.3	24 × 24	4.0/1.0	512 × 224	4
FSE/T1	ax	660	7.6	24 × 24	5.0/1.5	256 × 224	4
FSE/T1+fs	ax	680	7.6	24 × 24	5.0/1.5	256 × 224	4
STIR	ax	3620	53.2	24 × 24	5.0/1.5	256 × 192	2
3D/CUBE/T2	sag	3000	159	24 × 24	1.6/− 0.8	288 × 288	1
3D/LAVA	sag, ax, cor	4.2	2.0	40 × 36	4.0/− 2.0	320 × 192	0.7
DWI *b* = 1000	ax, sag	6000	93.1	42 × 42	8.0/2.0	128 × 128	8

## Review

### Scar/niche/other terms

**Fig. 2:**
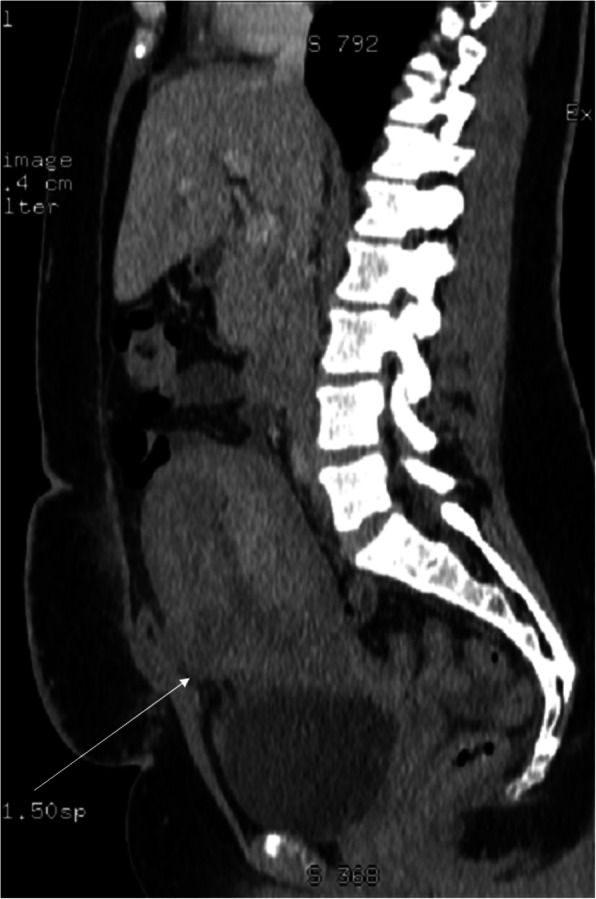
A 34-year-old primigravida primipara 12 days after C-section complicated by dissection of the posterior wall of the uterus. CT in a venous phase shows the normal postoperative appearance of a recent cesarean delivery incision which is hypodense as compared to the intact myometrium (thin arrow)

**Fig. 3:**
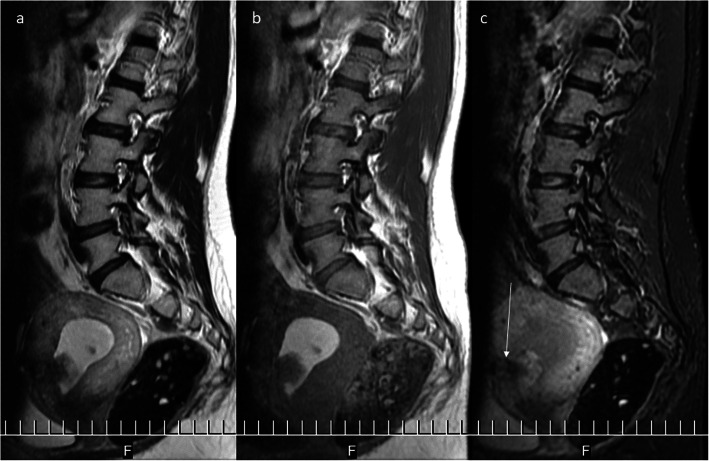
MRI of the lumbar-sacral part of the vertebral column in the sagittal plane. **a** FSE/T2WI. **b** FSE/T1WI. **c** STIR image. The study performed for neurological indications 2 weeks after C-section shows the forming scar in the anterior uterine wall, best appreciated as a thick hypointense band on STIR (**c** arrow). Blood (hyperintense on T1WI and T2WI, suppressed on STIR) and clot (T1- and T2-hypointense, slightly hyperintense on STIR) in a still distended uterine cavity

The cut of the uterus, like in any other surgical procedure, leads to scarring. If the patient requires imaging in the early post-Cesarean section period for some reason, we can observe the formation of the *scar*. If CT is performed for acute maternal indications, the healing uterine incision is seen as a hypodense part of the lower uterine segment, less enhancing than the remaining myometrium on post-contrast phases (Fig. 2). If MRI is performed for any reason, one can appreciate the signal changes in the myometrium of the lower uterine segment that depend on the time since C-section, reflecting the evolution of the blood in the incision site and forming scar which may present T2-hypointesity due to fibrous tissue (Fig. 3). If the uterine scar is incompletely healed, thinning and retraction of the uterine wall are observed with only residual myometrium adjacent to the scar. This forms a triangular or semicircular defect at the site of the scar which is T2-hyperintense and is called Cesarean scar *niche*. It has been defined as the indentation of the myometrium of at least 2 mm [8]. It has been reported that approximately 50% of women with a history of C-section have a uterine niche on hysterography, sonohysterography, or transvaginal ultrasonography (TVUS) [16]. The severity of complications has been related to niche size, and large niches are defined as having a depth of at least 50 or 80% of the anterior myometrium, or the remaining myometrial thickness ≤ 2.2 mm when evaluated by TVUS and ≤ 2.5 mm when evaluated by sonohysterography [17].

**Fig. 4:**
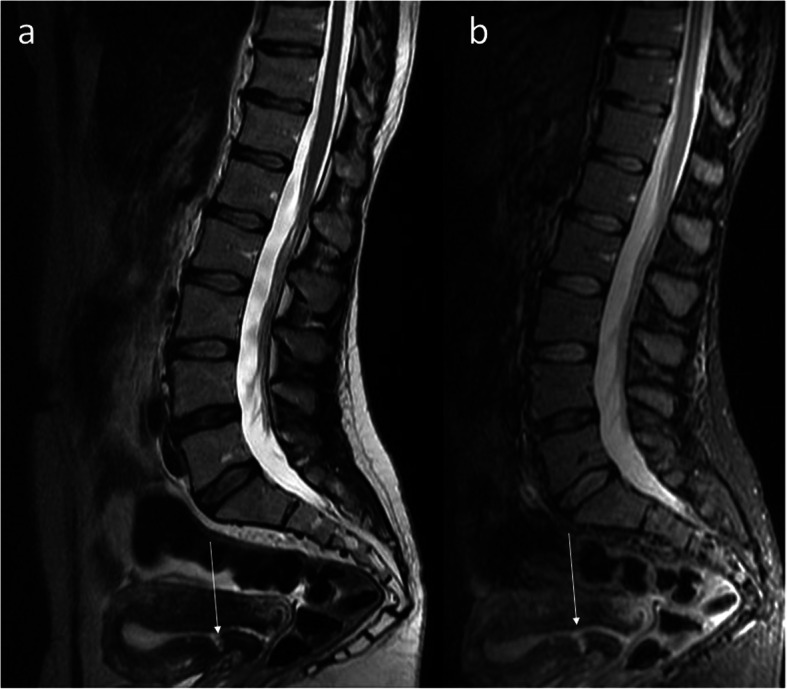
MRI of the lumbar-sacral part of the vertebral column in the sagittal plane. **a** FSE/T2WI. **b** STIR image. Post-C-section niche, hyperintense against the background of the hypointense myometrium, is shown by the arrows

**Fig. 5:**
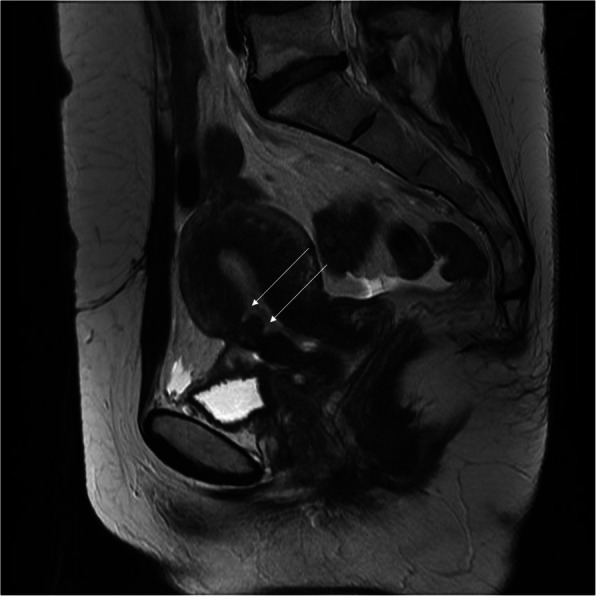
Pelvic MRI, FSE/T2WI in sagittal plane: two hyperintense niches after two C-sections (arrows)

The post-C-section niche belongs to the most frequent incidental findings on MRI of the lumbar-sacral part of the vertebral column which is much more frequently performed than pelvic MRI in women. The field of view (FOV) of this study quite often covers the uterus in part or even as a whole [18]. Extension of FOV of lumbar spine MRI in women generally seems to be a good idea, because in a significant percentage of cases, low back pain may have a gynecological cause as a consequence of the C-section for instance, but also of other pathologies of the female reproductive organs. The post-C-section niche presents as thinning of the myometrium in the anterior uterine wall above the cervix (Fig. 4a, b). After repeated C-sections, there may be two or more such defects. The abnormal outline of the uterine wall may be seen on both the internal and external sides of the scar (Fig. 5).

It is worth mentioning at this point that there is no general consensus in the literature as far as naming of changes in the uterus after C-section is concerned, with interchangeably used terms: *scar*, *scar defect*, *deficient cesarean scar*, *dehiscence*, *niche*, *isthmocele*, *pouch*, *diverticulum* [10, 11, 19, 20].

**Fig. 6:**
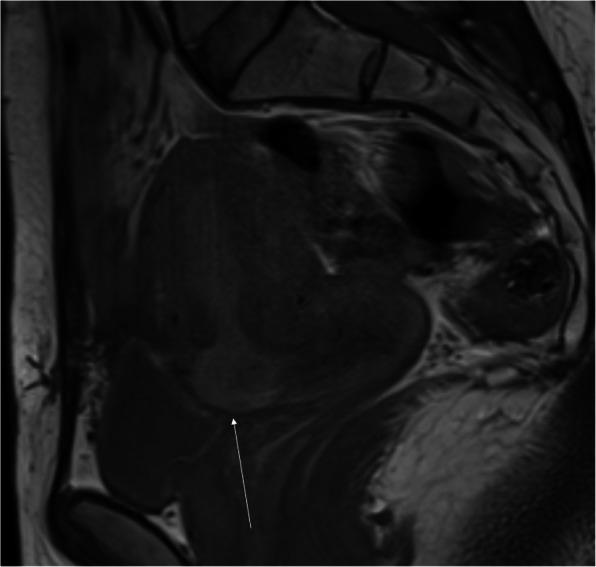
Pelvic MRI, SE/T1WI in the sagittal plane. Hyperintense blood reservoir in the C-section scar defect with intact serosa (arrow), which is isointense to the myometrium

The niche may be a reservoir of fluid or blood (hematoma soon after C-section or blood product accumulation in case of *adenomyosis* with T1-hyperintensity) (Fig. 6). Prolonged menstruation and abnormal postmenstrual bleeding are potential consequences. The niche may be a place of intrauterine device (IUD) malposition as well when the lower end of IUD is located in it, with—at least theoretical—risk of perforation [19].

### Dehiscence/rupture

**Fig. 7:**
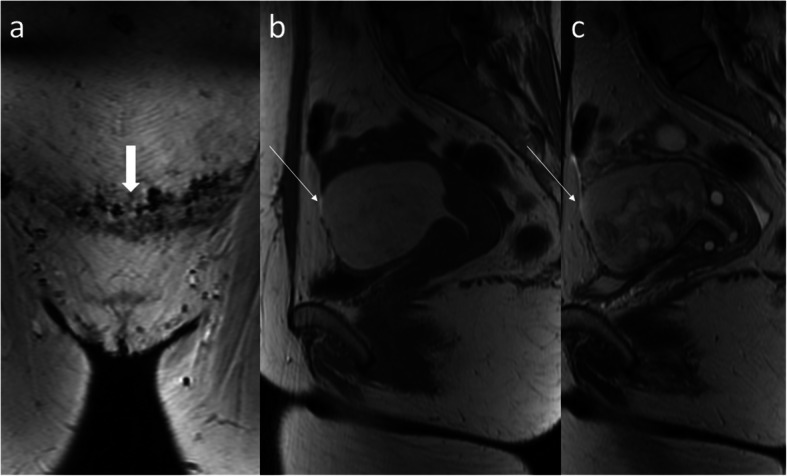
Pelvic MRI. A 42-year-old woman with a history of three C-sections. **a** GRE/T2*WI in the coronal plane (skin scar is shown by a thick arrow). **b** FSE/T1WI and **c** FSE/T2WI, both in the sagittal plane, depict a huge blood reservoir (T1-hyperintense, of mixed signal intensity on T2WI) with no serosal layer (no hypointense line) on the outer surface (thin white arrows). In pathology report: adenomyosis with autoamputation of the uterine body

If the endometrium and full thickness of the myometrium are ruptured, we face the incomplete rupture of the uterine wall that is called *uterine dehiscence*: only the serosal layer is intact in such cases and visible as a thin T2-hypointense line. It can lead to complete *uterine rupture* (or self-amputation of the uterine body) if the serosal layer is also torn. On MRI, there is no line on the periphery of the lesion that would separate the uterus from the surrounding tissues (Fig. 7). If it happens during pregnancy, uterine rupture requires immediate surgical intervention [11] and hysterectomy may be necessary although, if possible, a uterus-saving procedure is preferred.

### Adhesions

**Fig. 8:**
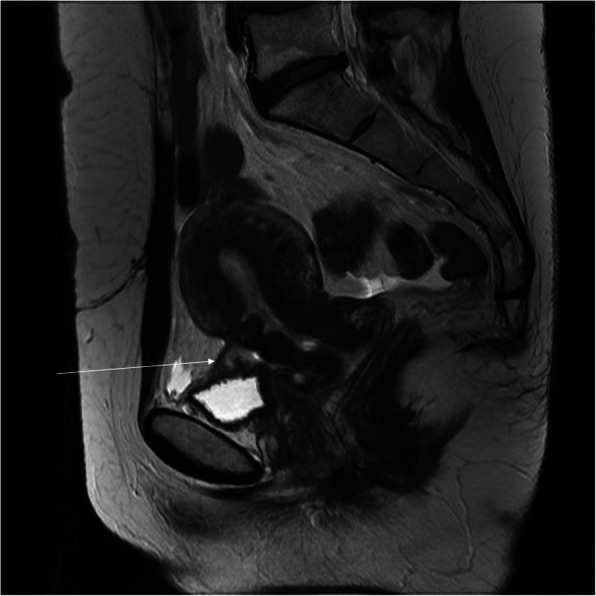
The same patient as in Fig. 5. The arrow points at T2-hypointense adhesion between the uterus and the bladder

**Fig. 9:**
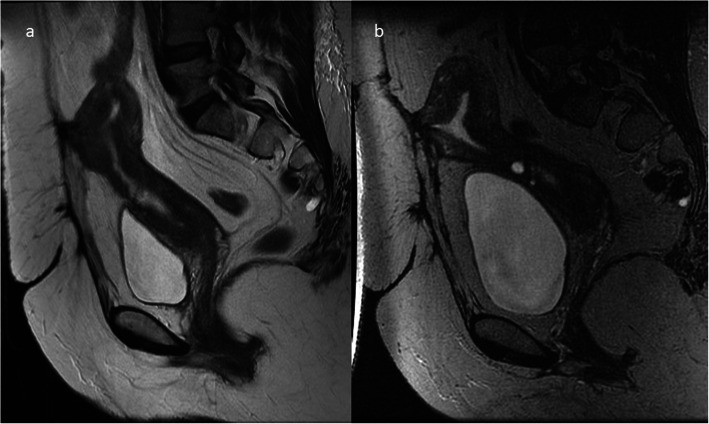
Pelvic MRI in the sagittal plane. FSE/T2WI (**a**). CUBE/3D/T2WI (**b**). Thick hypointense adhesions between the abnormally flexed uterus and an anterior abdominal wall

**Fig. 10:**
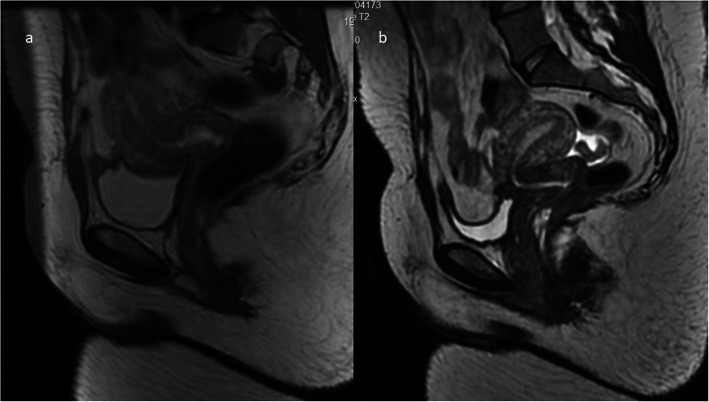
A 20-year-old woman with a history of synovial sarcoma of the right inguinal region, followed up routinely for oncological indications, 2 months (**a** FSE/T2WI) and 5 months (**b** CUBE/3D/T2WI) after C-section. The post-C-section scar is not visible; however, adhesions between the uterus and the bladder wall can be suspected (**a**) and are confirmed 3 months later, not only by the loss of the fat plane in between but also by the retroflexion of the uterus

As every surgical intervention, C-section may result in *adhesions*—both inside (*synechiae*) and outside the uterus. Similar to cases of endometriosis which implies the formation of adhesions, they can be directly visualized on MRI [21]. They present as T2-hypointense bands and after C-section and are most often and best seen between the anterior uterine wall and the bladder (Fig. 8) and between the uterus and the anterior abdominal wall (Fig. 9). The abnormal uterine position and flexion resulting from the adhesions (Fig. 10) may cause chronic pain and decreased fertility. Pelvic adhesions are also associated with other complications, including bowel obstruction and tubal obstruction. The latter, as well as uterine synechiae, may be another cause of infertility [11]. It is not infrequent that in a female patient with a history of C-section referred to lumbar spine MRI due to “low back pain,” the spine is normal and post-C-section niche and adhesions and/or endometriosis are detected.

### Endometrial implants

**Fig. 11:**
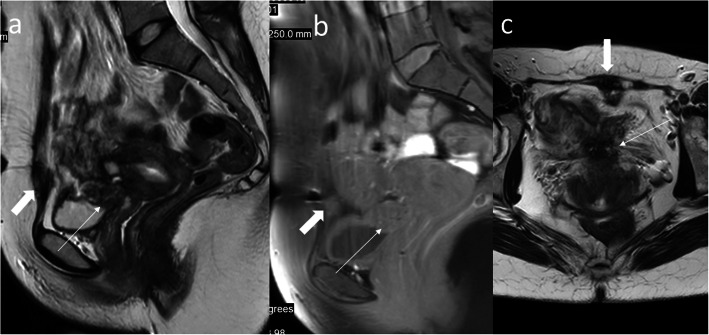
Pelvic MRI consulted for Sports Medicine Centre, Warsaw (images published with permission). FSE/T2WI in the sagittal (**a**) and axial (**c**) planes, FSE, T1 fat-saturated image, and the sagittal plane (**b**). Endometriotic implants on the way of C-section: in the bladder wall (thin arrows) and in the abdominal wall (thick arrows). The lesions are mostly T2-hypointense and T1-isointense, with very tiny foci of hyperintensity

*Endometrial implants* may be seeded on the way of surgical approach during C-section. MRI is a method of choice of their non-invasive detection [21]. Apart from adenomyosis that was mentioned before in the uterine wall itself, endometrial implants after C-section are most frequently seen in the anterior abdominal wall and in the bladder wall (Fig. 11). The foci of heterotopic endometrium in deep infiltrating endometriosis (DIE) are surrounded by hypertrophied fibrous-muscular tissue which results in the formation of solid nodules of low signal intensity on T2-weighted images. This is not always reflected in high signal intensity on T1-weighted images, or the lesions show only slightly increased signal intensity. The fat-saturated T1-weighted sequences are very important as they allow better visualization of small hyperintense endometriotic implants against a background without other hyperintense elements (fat) [21]. Even though the rates of scar endometriosis in the abdominal wall after C-section are reported as being up to 1.73% among women with endometriosis, Adriaanse et al., the authors of a large study of over 3000 women, suggest that the complication is underestimated (e.g., their study only described the incidence in women who underwent surgery, and not women with scar endometriosis who did not undergo surgery) and that with increasing rates of C-sections, the incidence will be higher [22]. Within the urinary system, the bladder is mentioned as a site most commonly affected by deep pelvic endometriosis (85% of cases). Up to 50% of patients with bladder endometriosis have a history of pelvic surgery with C-section in the first place [23]. Endometriosis is another cause of adhesions, pain, and infertility.

### C-section scar pregnancy

**Fig. 12:**
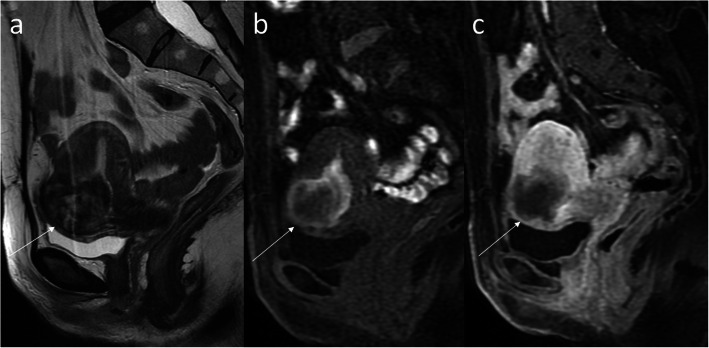
Pelvic MRI in the sagittal plane, FSE/T2WI (**a**). Dynamic contrast-enhanced fat-saturated T1 sequence before (**b**) and after (**c**) gadolinium administration. Retained products of conception (RPOC) of mixed signal intensity in the C-section niche after treatment with potassium chloride and methotrexate (arrows)

C-section scar may be a place of *ectopic pregnancy*. This ectopy is reported among the rarest forms of ectopic pregnancies, but up to 72% of C-section scar pregnancies occur in women with a history of 2 or more Cesarean deliveries [24]. After spontaneous abortion or termination of such pregnancy, the residual tissues of the placenta and decidua (retained products of conception (RPOC)) may be difficult to remove and require treatment with methotrexate. MRI may be necessary to assess the full extent of RPOC and in treatment monitoring. The signal intensity of RPOC may be variable on both T2- and T1-weighted images depending on the presence and degree of evolution of hemorrhage and on tissue necrosis. A heterogenous mass with contrast enhancement in the uterine wall and endometrial cavity in this clinical setting represents RPOC (Fig. 12) [25].

### Abnormal placentation

**Fig. 13:**
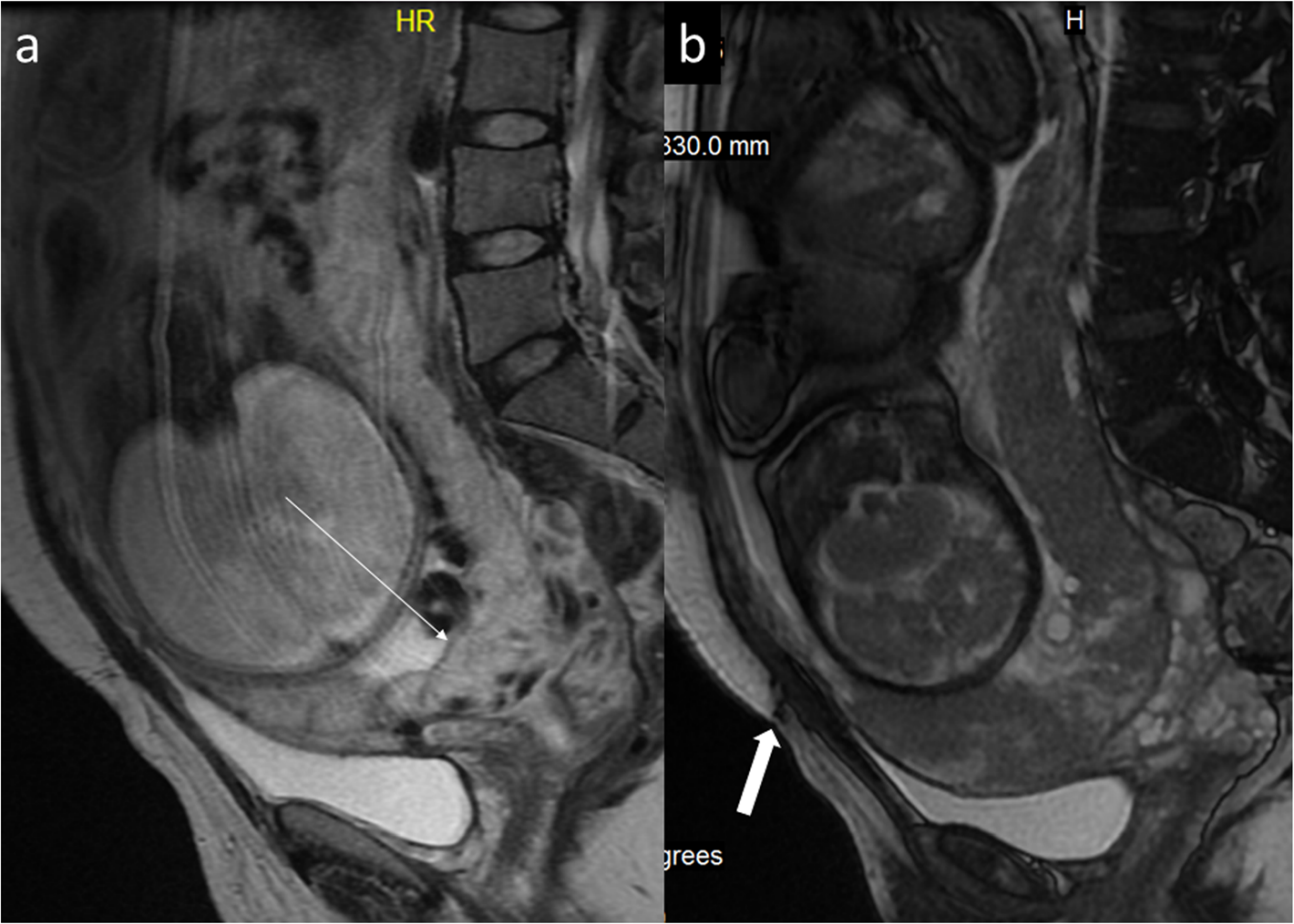
A 37-year-old woman, 4th pregnancy, 34 GW, history of 2 C-sections. FSE/T2WI (**a**) and FIESTA/2D image (**b**), both in sagittal projection, show placenta previa overlying completely the internal cervical os (**a**, thin arrow). The thick arrow points at the hypointense C-section scar in the abdominal wall (**b**)

**Fig. 14:**
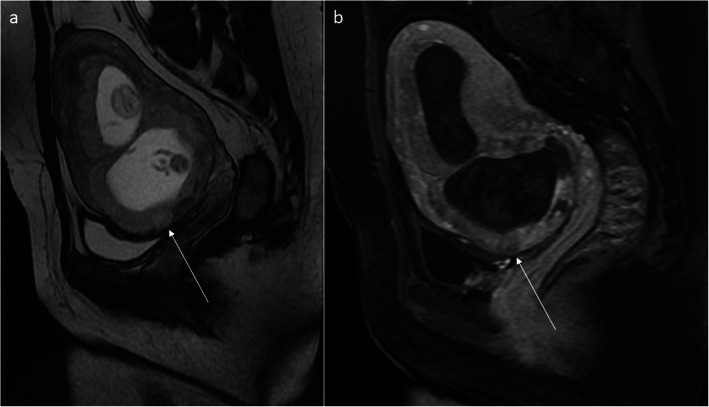
A 34-year-old woman, 4th pregnancy, dichorionic, diamniotic, 11 GW, history of 2 C-sections. FIESTA/2D image (**a**) and dynamic contrast-enhanced fat-saturated T1 sequence after gadolinium administration (**b**), both in sagittal projection, show placenta increta in the post-C-section scar. The urinary bladder did not show abnormalities on cystoscopy

**Fig. 15:**
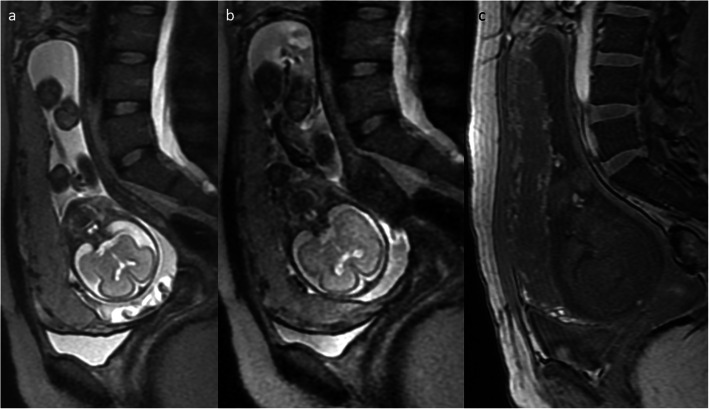
A 32-year-old woman, 3rd pregnancy, 24 GW, history of a classical C-section 2 years ago and ectopic scar pregnancy 1 year ago. FIESTA/2D image (**a**), SSFSE/T2WI (**b**), and FGR/T1WI (**c**), all in sagittal projection, show elevated vascularity at the placental-myometrial interface—placenta accreta in the post-C-section scar

**Fig. 16:**
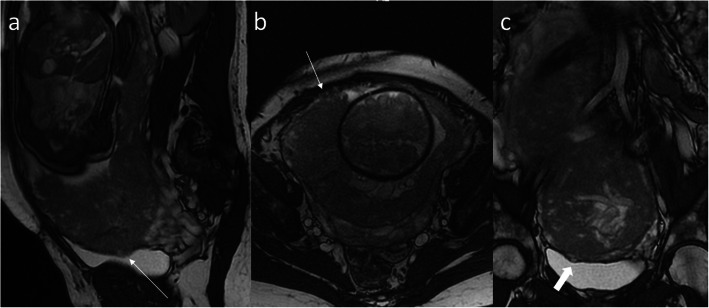
The same woman as in Fig. 13. The lack of visibility of the fragment of the bladder wall (thin arrow, **a**) and blurred placenta, uterus, and abdominal wall interface (thin arrow, **b**) are highly suggestive of placenta percreta while the bulge indenting the bladder with preserved bladder wall most likely represents the varix (thick arrow, **c**)

*Abnormal placentation* may also result from previous C-section which is the independent risk factor for placenta previa in the subsequent pregnancy (Fig. 13). Prior C-section and placenta previa are the two most important risk factors for placental adhesion disorder (PAD), also called morbidly adherent placenta (MAP), which includes placenta accreta, increta, and percreta [26, 27]. MAP can lead to placental retention, uncontrollable postpartum bleeding, postpartum infection, and hysterectomy although less definitive surgical intervention and/or uterine artery embolization is preferred, if possible [11]. MRI is recommended between 24 and 30 gestational weeks (GW), with a lower degree of diagnostic success before and after these gestational ages [28], although sometimes even an early MRI may show clearly the abnormality (Fig. 14). Gradient echo sequences enable delineation of the placental-myometrial interface while spin echo sequences depict T2-hypointense bands within the placenta that suggest invasive placentation (fortunately, there is no need to distinguish placenta accreta from placenta increta due to similar treatment). The study protocol should include single-shot fast spin echo T2-weighted sequences (vendor acronyms: HASTE, SSFSE, SSTSE, FASE) and balanced gradient echo sequences (TrueFISP, FIESTA, Balanced FFE, True SSFP, respectively) in the axial, sagittal, and coronal planes with respect to the uterus. The purpose is to visualize the entire placental-myometrial interface. T1-weighted images should be used to assess for retroplacental hemorrhage and DWI sequence—for invasion, in the best projection, most often sagittal [27, 28]. The cardinal imaging findings of placenta accreta are T2-hypointense intraplacental bands, heterogeneity of the placenta, and abnormal disorganized placental vascularity (Fig. 15) [29]. Diagnosis of placenta percreta is based on the lack of the myometrium and of the fat plane between the placenta and surrounding tissues with placental signal disrupting the T2-hypointense line of the bladder and/or bowel wall, or abdominal wall muscles [28]. However, both in the literature and in the author’s own experience, there are a number of pitfalls that must be taken into account while using MRI as an adjunct to US in the diagnostic process of abnormal placentation. Possible pitfalls include the following:

- Thinning or even loss of retroplacental T2 dark zone which may be a normal finding in the growing pregnancy or may be encountered in case of uterine dehiscence after previous C-section
- Blood clots mimicking dark intraplacental bands
- Bulges indenting the bladder that suggest invasion while they may represent bladder varices (Fig. 16) or focal bulge in the region of the maternal umbilicus which is a physiological finding caused by the separation of the rectus muscles in growing pregnancy [26, 27]

All the authors in the literature stress the necessity of complementary use of both US and MRI in these clinical situations [26–29].

## Summary

Magnetic resonance imaging provides comprehensive anatomical insight into the pelvis and its pathology facilitating clinical decisions thanks to its higher tissue resolution and a bigger field of view than ultrasound. The sequelae of Cesarean section can be depicted on lumbar spine MRI in a certain percentage of cases as an incidental finding and corroborated in detail on targeted pelvic MRI in order to cope with complications.

## Data Availability

Data sharing is not applicable to this article as no new data were created or analyzed in this study.

## Abbreviations

C-section: Cesarean section
CT: Computed tomography
GW: Gestational weeks
IUD: Intrauterine device
LSCS: Lower (uterine) segment cesarean section
MAP: Morbidly adherent placenta
MRI: Magnetic resonance imaging
NICE: National Institute for Health and Care Excellence
OECD: Organisation for Economic Co-operation and Development
PAD: Placental adhesion disorder
RPOC: Retained products of conception
T1WI: T1-weighted image
T2WI: T2-weighted image
TVUS: Transvaginal ultrasonography
US: Ultrasonography
WHO: World Health Organization
